# A multifactorial approach of nutritional, intellectual, brain development, cardiovascular risk, socio-economic, demographic and educational variables affecting the scholastic achievement in Chilean students: An eight- year follow-up study

**DOI:** 10.1371/journal.pone.0212279

**Published:** 2019-02-20

**Authors:** Daniza M. Ivanovic, Atilio F. Almagià, Violeta C. Arancibia, Camila V. Ibaceta, Vanessa F. Arias, Tatiana R. Rojas, Ofelia C. Flores, Francisca S. Villagrán, Liliana U. Tapia, Javiera A. Acevedo, Gladys I. Morales, Víctor C. Martínez, Cristián G. Larraín, Claudio F. A. Silva, Rodrigo B. Valenzuela, Cynthia R. Barrera, Pablo B. Billeke, Francisco M. Zamorano, Yasna Z. Orellana

**Affiliations:** 1 Laboratory of Nutrition and Neurological Sciences, Human Nutrition Area, Institute of Nutrition and Food Technology Dr. Fernando Monckeberg Barros (INTA), University of Chile, Santiago, Chile; 2 Laboratory of Physical Anthropology and Human Anatomy, Institute of Biology, Faculty of Sciences, Pontifical Catholic University of Valparaíso, Valparaíso, Chile; 3 Center for Research in Education and Learning, University of Los Andes, Santiago, Chile; 4 School of Nutrition and Dietetics, Faculty of Medicine, Andres Bello University, Santiago, Chile; 5 School of Nutrition, Faculty of Medicine, University of Costa Rica, San José, Costa Rica; 6 Public Health Department, Faculty of Medicine, University of La Frontera, Temuco, Chile; 7 Department of Commercial Engineering, Faculty of Economics and Business, University of Chile, Santiago, Chile; 8 Radiology Department, Faculty of Medicine-German Clinic of Santiago, University of Development, Santiago, Chile; 9 Department of Nutrition, Faculty of Medicine, University of Chile, Santiago, Chile; 10 Division of Neuroscience, Center for Research in Social Complexity (neuroSIS), Faculty of Government, University of Development, Santiago, Chile; 11 Advanced Quantitative Imaging Unit, Image Department, German Clinic of Santiago-University of Development, Santiago, Chile; Cairnmillar Institute, AUSTRALIA

## Abstract

The aim of this study was to quantitate the relative impact of nutritional, intellectual, brain development, cardiovascular risk, socio-economic, demographic and educational variables on the results of the 2009 Quality Education Measurement System (SIMCE) tests of language and mathematics for scholastic achievement (SA) applying a multifactorial approach, in school-age children of the 2010 5^th^ elementary school grade (5ESG) and of the 1^st^ grade of high school (1HSG). The purposes were: i) to test the hypothesis that intellectual ability, the level of SA of the educational establishments in the 2009 SIMCE tests, sex, parental schooling levels, and head circumference-for-age Z-score are the most relevant parameters associated with 2009 SIMCE outcomes; ii) to determine the predictive ability of the 2009 SIMCE results in determining the 2013 SIMCE outcomes for the 2010 5ESG cohort (when they graduated from elementary school, 8^th^ grade) and for determining the 2013 University Selection Test (PSU) outcomes for the 2010 1HSG group (for university admission, when they graduated from high school, 4^th^ grade); iii) to determine the association between the 2009 SIMCE results with the 2017 PSU outcomes for the 2010 5ESG group (for university admission, when they graduated from high school, 4^th^ grade). A representative, proportional and stratified sample of 33 schools of the Metropolitan Region of Chile was randomly chosen. In these schools, 1,353 school-age children of both sexes, of the 2010 5ESG (n = 682; mean age = 10.8 years, SD = 0.6) and of the 2010 1HSG (n = 671; mean age = 14.8 years, SD = 0.6) participated. In both grades and tests, the findings confirm the hypotheses formulated. 2009 SIMCE outcomes were positively and significantly associated with 2013 SIMCE and with 2017 PSU and, with 2013 PSU outcomes in school-age children from 2010 5ESG and 1HSG, respectively. These findings may be useful for educational and health planning in Chile and countries in a comparable stage of development.

## Introduction

One of the main national priorities in Chile is the improvement of the quality of the educational process. However, this is a multifactorial problem that depends on particular qualities of the child, his/her family and numerous factors related to the educational system such as the teachers’ academic background, their teaching methodologies and the infrastructure of the educational establishments [1–9].

All the factors mentioned above affect greatly the development of countries which is hampered by shortcomings in educational, socio-economic, socio-cultural, intellectual and nutritional conditions which affect the quality of life of their population. Few investigations have been carried out in Chile attempting to quantitate the relative impact of these factors on educational outcomes by applying standardized tests with national coverage. For instance, the long-term effects of nutritional status at an early age on scholastic achievement (SA) has not been sufficiently defined; additionally, research with a global approach of the predictors mentioned previously towards the follow-up of these studies is even scarcer [4,5,7–12]. Poor school-age children tend to become poor adults; this may be the expression of unsatisfactory educational outcomes. This may be explained by prevailing deficient home conditions and negative environments which do not stimulate adequately the learning processes, the development of intellectual capabilities and the fostering of health self-care [5,7,12–16].

Educational inequalities result from the extent to which students with different socio-cultural origins have unequal probabilities of attaining satisfactory SA levels; as a consequence, cognitive abilities of children are very different across countries [17–19]. Other authors have proposed that variables related to the family’s socio-economic status (SES) such as paternal schooling, and in particular maternal schooling, are consistent in explaining child SA, intelligence, and nutritional status, probably because mothers are the main source of intellectual stimulation and enrichment in the psycho-social environment and the health-related behaviour of the family [4,5,7,11,12,20–24]. Besides maternal schooling, the family structure and stability and prenatal parental smoking have also been identified as important predictors of SA and child intelligence [25,26]. Other significant aspects that favour better SA arise from the presence of the father at home, the family size and the number of siblings [27,28]; however, other authors suggest that the apparent birth-order effect on SA or intelligence is an artefact of family size or that it is non-significant [29,30]. Studies by other authors report that sex differences in language and mathematics performance generally favour females and males, respectively, although recent data suggest that in many countries this gap is closing [31–33].

Early anthropometric and nutritional measurements such as birth weight, birth length and breastfeeding are considered of great importance for later nutritional status and cognition [10,11,20,34–40]. Our previous findings reveal that nutritional status is positively and significantly associated with socio-economic indicators, SA, dropout, intelligence, and brain development [2–7,11,13,20, 21,35]. Indicators of past nutrition (more than prenatal nutritional background and early nutritional measurements indicators), such as weight, height and head circumference, the anthropometric indicator of nutritional background and brain development, are of considerable importance during school age since nutrition affects both SA and intelligence [2–7,11–14,20,41,42]; moreover, school dropout relates with head circumference but not with weight or height [41]. In addition, several publications show that obesity and physical fitness could be important factors not only for health but also for SA [4,5,12,43–48].

The child’s intelligence represents the most relevant parameter that explains SA and is significantly associated with maternal schooling, maternal intelligence, head circumference and brain volume as well as with the antecedent of undernutrition in the first year of life [2,3,7,11–14,20,21,34–36,42,49,50]. This is how the findings from several authors confirm that differences in human brain size are relevant in explaining differences in SA and intelligence [2–7,11–14,20,35,36,41,42,50,51]. As a consequence, children older for the school grade have significantly lower SA and intelligence parameters, deprived socio-economic conditions and lower level of education and occupation of parents, decreased brain measurements, suboptimal nutritional status and higher repetition and dropout rates [4,21,34]. Other results verify that SA and intelligence markers in girls are inversely and significantly associated with age at menarche [52].

In Chile, the quality of school education is evaluated by the Quality Education Measurement System (SIMCE) tests administered by the Agency for Education Quality, for elementary and high school students and by the University Selection Test (PSU), which evaluate the quality of education at the end of the high school cycle. These standardized tests have nationwide coverage [53–55].

The full expression of the genetic potential of school- age children depends on multiple environmental factors by which, the educational process presents itself as a multifactorial phenomenon. In this context, SA will be the result of the interaction of child, family and educational system factors and children with cognitive, behavioural and educational problems must be detected at an early age [56]. This can be accomplished by adequate diagnoses which must be formulated to institute efforts for its evaluation and referral, if necessary, to improve their outcomes [57].

Building on this existing literature, the aim of this study was to quantitate the relative impact of nutritional, intellectual, brain development, cardiovascular risk, socio-economic, demographic and educational variables on the results of the 2009 SIMCE tests of language and mathematics. In this research, the impact of these variables on SA was carried out applying a multifactorial approach, in school-age children of the 2010 5^th^ elementary school grade (2010 5ESG) and of the 1^st^ grade of high school (2010 1HSG). The purposes were: i) to test the hypothesis that intellectual ability, the level of SA of the educational establishments in the 2009 SIMCE tests, sex, parental schooling levels, and head circumference-for-age Z-score are the most relevant parameters associated with 2009 SIMCE outcomes; ii) to determine the predictive ability of the 2009 SIMCE results in determining the 2013 SIMCE outcomes for the 2010 5ESG cohort (when they graduated from elementary school, 8^th^ grade) and for determining the 2013 PSU outcomes for the 2010 1HSG group (for university admission, when they graduated from high school, 4^th^ grade); iii) to determine the association between the 2009 SIMCE results with the 2017 PSU outcomes for the 2010 5ESG group (for university admission, when they graduated from high school, 4^th^ grade).

## Material and methods

### Study design

This is an observational, cross-sectional and follow-up study.

### Study population

The target population, 187,860 children (39% of the Chilean school population), included all school-age children enrolled in the 5ESG (*N* = 91,663) and in the 1HSG *(N =* 96,197) in the Metropolitan Region of Chile in 2010 who took the SIMCE tests at the end of November 2009. They belonged to public, private-subsidized, and private non-subsidized schools from urban areas [58].

### Sample selection

**Fig 1:**
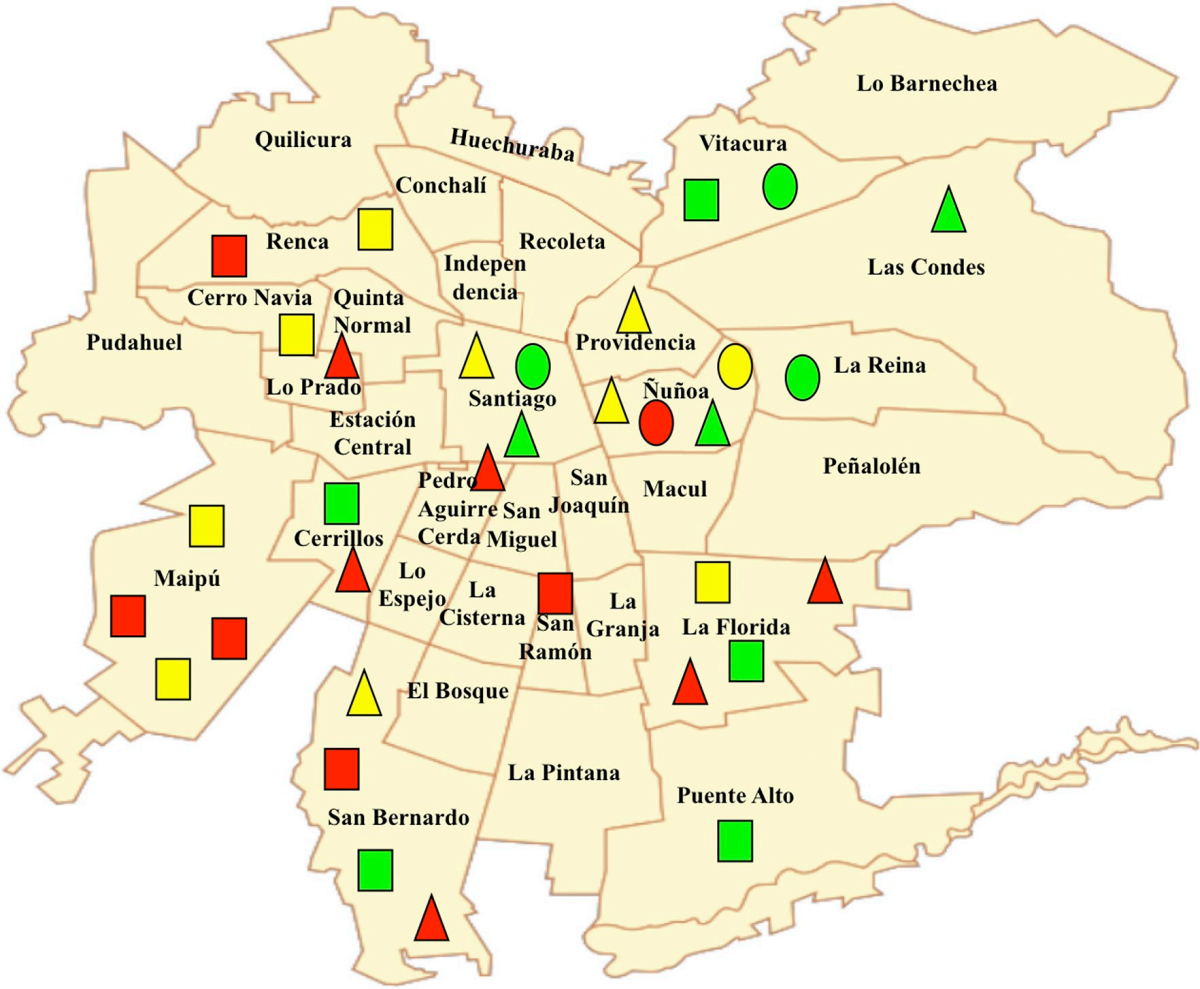
Geographical distribution in the Metropolitan Region of Chile, of the educational establishments chosen in the sample with high (green), medium (yellow) and low (red) level of scholastic achievement in the 2009 SIMCE tests by type of school: Public (triangle); private subsidized (square); private non-subsidized (circle). *n* = 33.

At the onset of March 2010, the Agency for Quality Education from the Ministry of Education provided us the results of the 2009 SIMCE tests for each school-age children. These results were stratified according to type of school (public, private-subsidized, and private non-subsidized), level of SA of educational establishments in the 2009 SIMCE tests (high, medium and low) and sex. Population sampling was then performed to target a representative sample of students from the educational establishments from the urban areas of the Metropolitan Region of Chile. The sampling was carried out as follows: firstly, 33 educational establishments which represented 2.61% of the total population of the urban schools (*N* = 1,262), were randomly selected by proportional allocation according to their stratification by type of school and level of SA of the educational establishments in the 2009 SIMCE tests and identified by: green (high), yellow (medium) or red (low) markers (Fig 1).

Secondly, in each of these 33 schools all students enrolled in both grades who took the 2009 SIMCE as well as their parents, the principals of the establishments and the language (L) and mathematics (M) teachers were invited to participate. A total of 1,353 school-age children of the 2010 5ESG (*n* = 682; males *n* = 379) and of the 2010 1HSG (*n* = 671; males *n* = 330), (95% of the original sample), as well as their parents, the school principals and teachers, agreed to participate and signed the informed consent form. Students’ ages ranged from 9.9y to 18.2y (mean 10.8; SD = 0.6y) and from 12.7y to 17.6y (mean = 14.8; SD = 0.6y), in the 2010 5ESG and in the 2010 1HSG, respectively.

### Procedures of the field study

When Grant FONDECYT 1100435 was approved: i) the first part of the field study was carried out from March to September 2010, in order to collect data of the 2009 SIMCE outcomes and to assess the nutritional, intellectual, brain development, cardiovascular risk, socio-economic, demographic and educational variables. When Grant FONDECYT 1150524 was approved, the second part of the field study was carried out: ii) from March to May 2015, with the purpose to register, four years later, the 2013 SIMCE outcomes of the 2010 5ESG students who graduated from elementary school on 2013 and took the 2013 SIMCE tests and, the 2013 PSU outcomes of the 2010 1HSG students who graduated from high school on 2013, and took the PSU; and iii) eight years later, during June 2018, in order to register the 2017 PSU outcomes of the 2010 5ESG students who graduated from high school on 2017.

### Ethical considerations

This study was approved by the Committee on Ethics in Studies in Humans of the Institute of Nutrition and Food Technology Dr. Fernando Monckeberg Barros (INTA), University of Chile, and ratified by the Committee on Bioethics of the National Fund for Scientific and Technological Development (FONDECYT), Chile. During 2010 and 2015 subjects' consent was obtained according to the norms for Human Experimentation, Code of Ethics of the World Medical Association (Declaration of Helsinki) [59].

### Data collection conducted during 2010

#### 2009 SIMCE outcomes

SA was assessed through the 2009 SIMCE tests, which has national coverage and is administered by the Agency for Education Quality. The aim of this testing was to evaluate changes in the quality and equitableness of the educational process in the different areas covered by the national curriculum. Scores range between 0 and 400 and the results are expressed as mean ± SD in the L (LSA) and the M (MSA) SA tests. Data were also categorized in three ranges of achievements: high, medium and low in relation to the SA of the school-age children and the SA of the educational establishments, as defined by the Ministry of Education for these grades [53].

#### Nutritional status

Prenatal nutritional background and early nutritional measurements such as birth weight, birth length and duration of breastfeeding were reported by the mothers who presented the document that was delivered to them by the hospital registry where their children were born. Measurements of weight, height and head circumference were carried out at school using standardized procedures; all the instruments were verified before measuring each subject [60]. The postnatal nutritional status was expressed as height-for-age Z-score according to NCHS-CDC tables [61,62]. Head circumference-for-age Z-score was assessed using the tables of D. Ivanovic, Olivares, Castro, and Ivanovic, 1995 [63], Nellhaus, 1968 [64,65], Roche, Mukherjee, Guo, and Moore, 1987 [66] and Tanner, 1984 [67]. Head circumference-for-age Z-score values were similar when applying these four tables (correlation coefficient between these patterns 0.98) [63]. The current nutritional status was expressed as body mass index (BMI, weight/height^2^) and compared to the NCHS-CDC tables and expressed as body mass index Z-score (Z-BMI) [61,62].

BMI was calculated using biological age derived from the Tanner stages [67]. Birth weight and birth length were used as indices of prenatal nutrition, height-for-age Z-score and head circumference-for-age Z-score served as indicators of postnatal nutrition and BMI was used as an index of current nutritional status.

#### Intellectual ability

Intellectual ability was assessed with the standard version of the Raven's Progressive Matrices Test in book form, with a general scale for children 12 years or above that had been standardized for Chilean school-age children [68,69]. The test was administered collectively in the classroom by an educational psychologist and scores were recorded in a percentile scale according to age, with the following grading: Grade I = superior intellectual ability; Grade II = above average; Grade III = average; Grade IV = below average and Grade V = intellectually defective. WHO experts on its application in developing countries have recommended Raven’s test because its results are not affected by culture [70].

#### Brain development

Brain development was assessed through the measurement of head circumference and expressed as Z-score for-age, as previously stated. Head circumference measurement is a simple method to assess brain growth and has been defined as an anthropometric indicator of both nutritional background and brain development [49,60,71].

#### Cardiovascular risk factors

In addition to BMI waist circumference was measured using standard procedures and abdominal obesity was calculated using a percentile scale [72]. Systolic and diastolic blood pressures were classified as normal or high in a percentile scale [73]. Diabetes and smoking (number of cigarettes per day) were also registered. The cardiovascular risk index was expressed into a single score as number of risk factors and they were also considered individually.

#### SES

SES was measured applying a score based on Graffar's modified parameters which consider schooling and occupation of the household head, and characteristics of the housing (building materials, ownership, water supply and ownership of durable goods) [74]. Graffar's modified scale has been adapted for Chilean urban and rural populations and classifies the population into five socio-economic strata: 1 = high; 2 = medium-high; 3 = medium; 4 = medium-low; and 5 = low.

#### Demographic variables

Some demographic characteristics such as the student’s age, sex, age of menarche, number of siblings, order of place among siblings, number of family members, crowding (persons/bedroom) and promiscuity (persons/bed) were registered.

#### Variables dependent on the educational system

Teachers were invited to answer a questionnaire that evaluates their academic background and teaching methodologies. An index was calculated for these variables, which finally was expressed as scores and categorized as adequate, regular and deficient. The principals of the schools were interviewed to assess the infrastructure of school. This information was confirmed by the investigators and was expressed as scores categorized as adequate, regular and deficient. Other educational variables considered were the type of school and the level of SA of the educational establishment in the 2009 SIMCE tests.

## Follow-up study

### Data collection conducted during 2015

#### 2013 SIMCE outcomes

Four years later, during 2013, 2010 5ESG school-age children were in the 8^th^ grade of elementary school and graduated from elementary school. They took the 2013 SIMCE on November of this year both LSA and MSA scores and data were also categorized as we explained previously [53]. The aim was to establish whether the 2009 and 2013 SIMCE outcomes were interrelated.

#### 2013 PSU outcomes

Four years later, during 2013, 2010 1HSG school-age children were in the 4^th^ grade of high school and graduated from high school. They took the 2013 PSU, on November of this year. LSA and MSA scores in the 2013 PSU were also registered and these data were provided by the Department of Evaluation, Measurement and Educational Registry (DEMRE) of the University of Chile and, by the Studies Centre of the Ministry of Education during 2015 and were also graded as follows both in LSA and MSA tests: low SA (< p25; score < 450), medium SA (≥ p25 and ≤ p 75; 450–620) and high SA (> p75; > 620). In fact, scores below 450 barred students from applying to higher education [54]. The purpose was to determine to what extent SA in the 2009 SIMCE would predict PSU outcomes four years later. The PSU, the baccalaureate examination with national coverage for admission to university, has minimum and maximum scores of 150 and 850, respectively. The L test considers 90 items and the M test 60 [54].

### Data collection conducted during 2018

#### 2017 PSU outcomes

Eight years later, at the end of November 2017, 2010 5ESG school-age children were in the 4^th^ grade of high school and graduated from high school and they took the 2017 PSU, both LSA and MSA scores. L and M tests consider 80 items each. LSA and MSA scores in the 2017 PSU were provided by the Department of Evaluation, Measurement and Educational Registry (DEMRE) of the University of Chile and, by the Studies Centre of the Ministry of Education at the onset of June 2018, as part of the development of Grant FONDECYT 1150524. [55]. Results were expressed in the same categories of the 2013 PSU.

#### Statistical analysis

Data analysis included chi-squared test for ordinal variables and ANOVA for comparison of means. Pearson and Spearman correlation coefficients were used for quantitative and ordinal variables, respectively. In the linear regression analysis, the stepwise procedure was used to establish the most important independent variables that could affect LSA and MSA in the SIMCE tests (dependent variable). The determination coefficient (*R*^*2*^) was calculated to measure the fit of the regression models [75]. For all hypothesis tests the level of significance was 0.05. Data were processed using the software Stata 14.

## Results

### Distribution of the sample according to SES

The distribution of the sample according to SES categories was as follows: 0.3% belonged to high SES, 14.7% to medium-high SES, 38.3% to medium SES, 45.6% to medium-low SES and 1.1% to low SES.

### SIMCE outcomes according to sex

In school-age children from the 2010 5ESG, LSA and MSA did not differ between males and females. However, in the 2010 1HSG, males achieved higher LSA scores (286.1 ± 48.6 (*n* = 330)) than females (274.1 ± 49.70 (*n* = 341)); (*F* = 8.35; *p* = .0040) and the same was observed for MSA scores (308.3 ± 50.7 (*n* = 330)) and (277.1 ± 51.6 (*n* = 341)), respectively (*F* = 52.75; *p* < .0001).

### Distribution of the sample by type of school attended and the level of SA of the educational establishment

**Fig 2:**
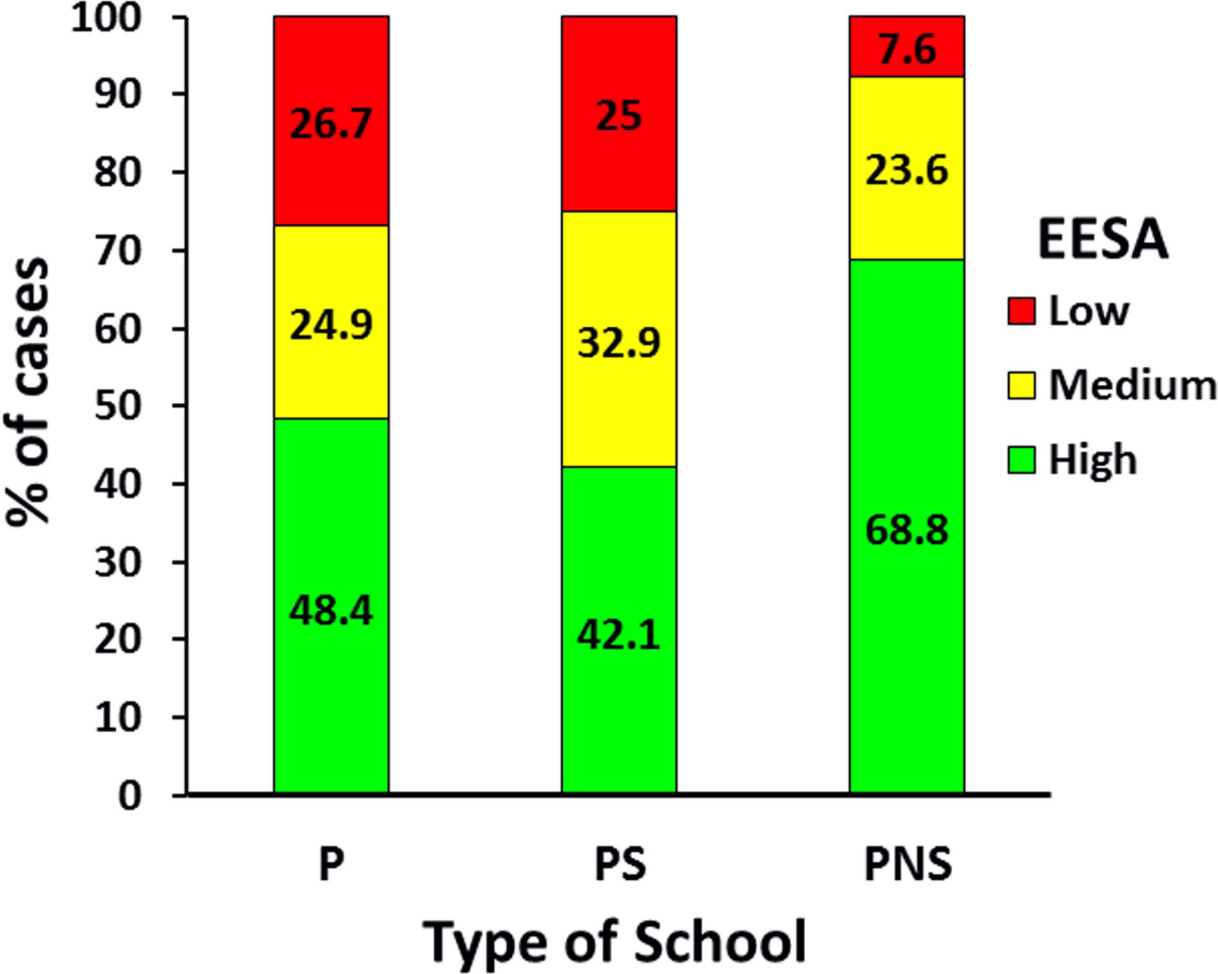
Distribution of the sample by the level of scholastic achievement of the educational establishments (EESA) in the 2009 SIMCE tests and type of school. *Note*. P = public schools; PS = private subsidized schools; PNS = private non-subsidized schools. (*n* = 1,353). *X*^*2*^_*o*_ (4df) = 36.381 > *X*^*2*^_*t*_ (4df) *p <* .001 = 18.465.

Differences were found in the distribution of students depending on the type of school attended and the level of SA of the educational establishments since in the private non-subsidized schools group most of the school-age children were attending to educational establishments with high SA (68.8%) compared with public schools (48.4%) and private-subsidized schools (42.1%) (Fig 2; *p <* .001).

### Associations between the level of SA of the educational establishments, SES and type of school

**Fig 3:**
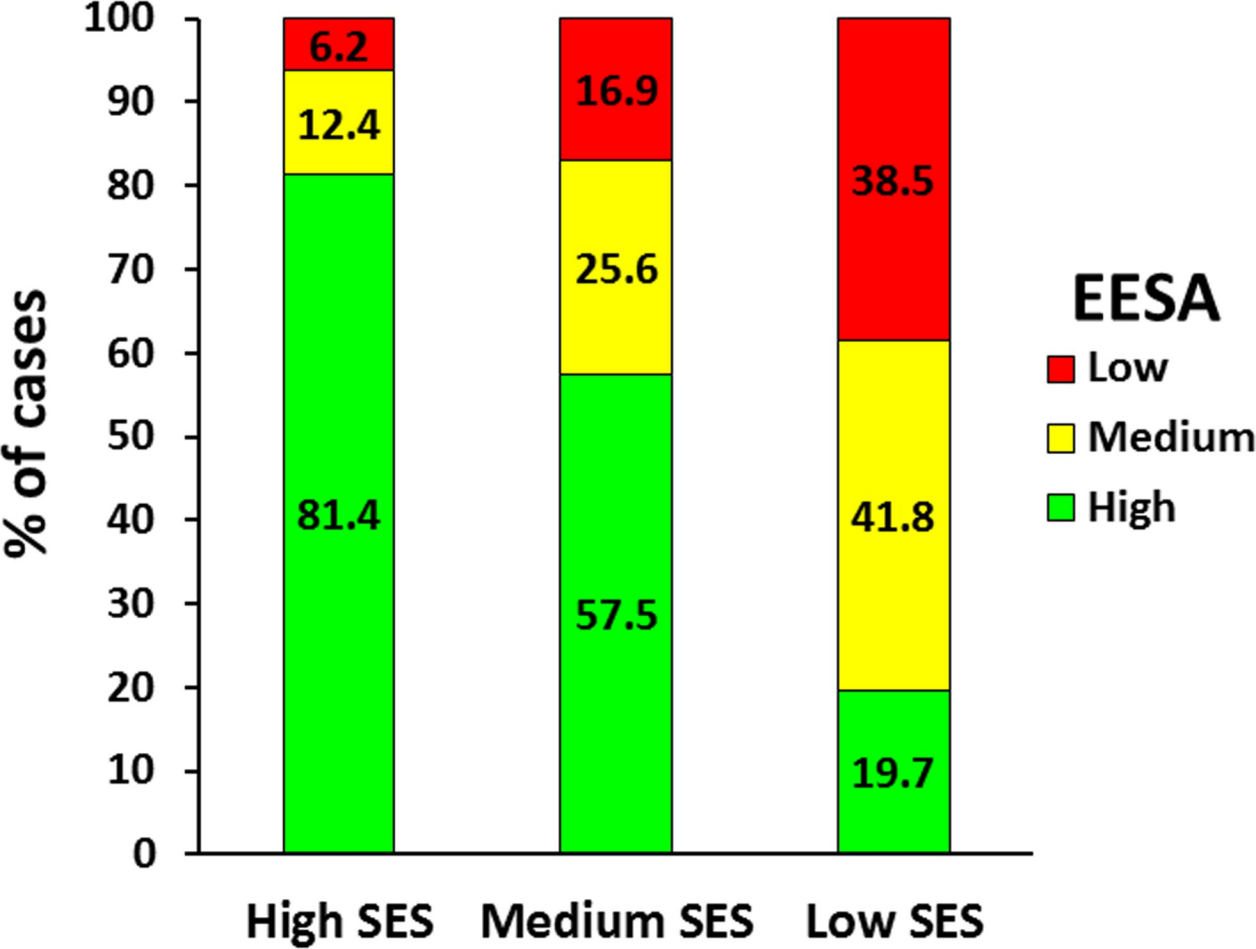
Distribution of the sample by the level of scholastic achievement of the educational establishments (EESA) in the 2009 SIMCE tests and socio-economic status (SES). (*n* = 1,353). *X*^*2*^_*o*_ (4df) = 291.256 > *X*^*2*^_*t*_ (4df) *p <* .001 = 18.465.

**Fig 4:**
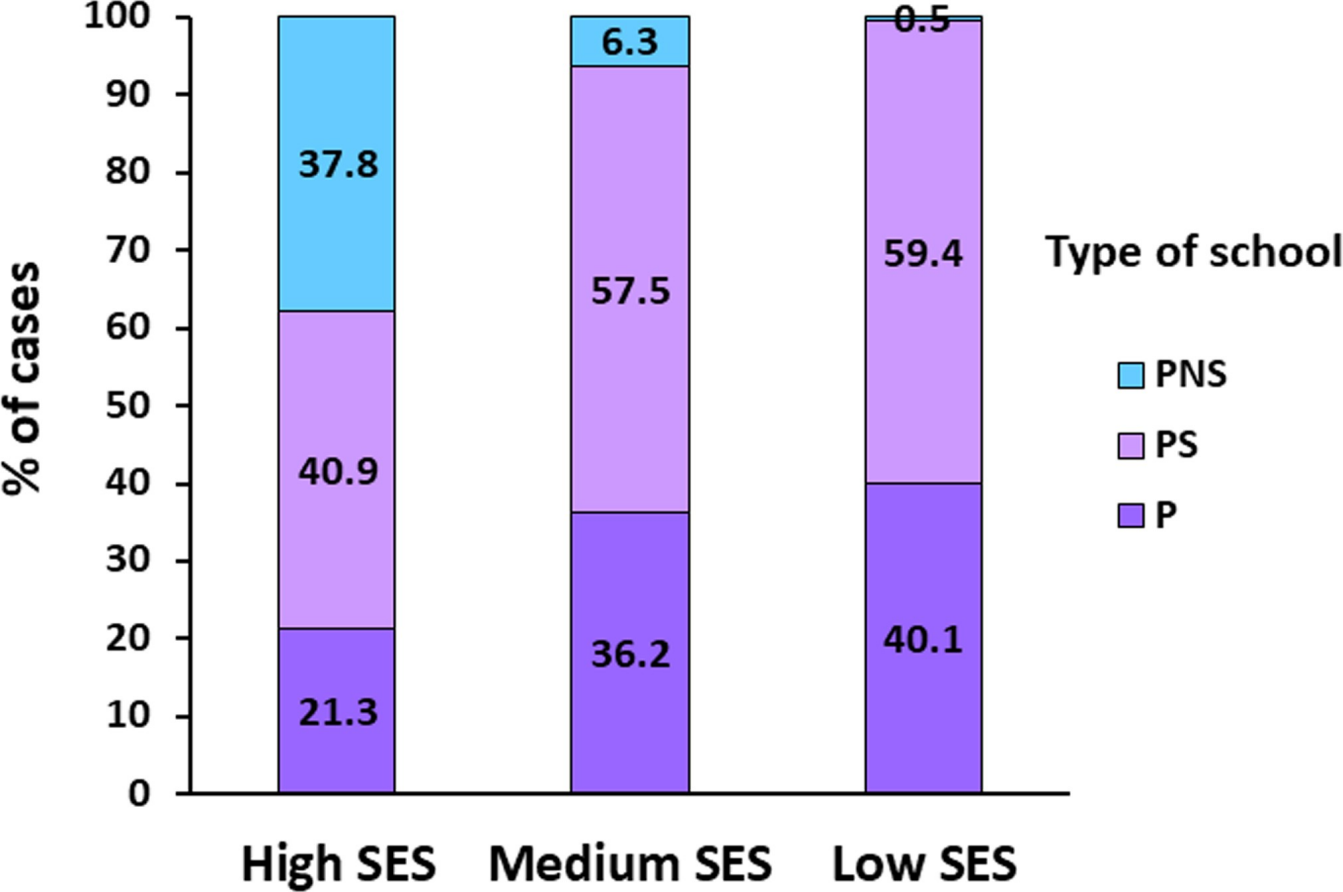
Distribution of the sample by type of school and socio-economic status (SES). *Note*. P = public; PS = private subsidized; PNS = private non-subsidized. (*n* = 1,353). *X*^*2*^_*o*_ (4df) = 268.808 > *X*^*2*^_*t*_ (4df) *p <* .001 = 18.465.

SA level of the educational establishments in the 2009 SIMCE tests was also positively associated with SES (Fig 3; *p* < .001) and SES significantly differed according to type of school attended (Fig 4).

### Correlation coefficients between the 2009 SIMCE outcomes both LSA and MSA (dependent variables) and psychological, nutritional, cardiovascular risk, socio-economic, socio-cultural, demographic, family, and educational factors (independent variables) by grade

Table 1 shows the correlation coefficients between LSA and MSA in the 2009 SIMCE tests (dependent variables) and independent variables by school grade. Intellectual ability positively correlated with 2009 SIMCE outcomes in LSA and MSA in 2010 5ESG (*p* < .0001). Prenatal nutritional background and early anthropometric measurements such as birth weight and birth length did not correlate with 2009 SIMCE outcomes; the exception was the duration of breastfeeding in MSA of 2010 5ESG (*p* = .0364). Postnatal nutritional background markers height-for-age Z-score and head circumference-for-age Z-score, correlated positively and significantly with the 2009 SIMCE outcomes in both grades and tests. The correlations being higher for MSA than LSA, especially for head circumference-for-age Z-score in school-age children from both grades (*p* < .0001). A negative correlation between the 2009 SIMCE and current nutritional status expressed as Z-BMI was found only for LSA in school-age children from the 2010 1HSG (*p* = .0019). With regard to cardiovascular risk factors, negative and significant correlations were found only in the 2010 1HSG for both tests, with the exception of tobacco use for LSA in school-age children from the 2010 1HSG. Positive and significant correlations between 2009 SIMCE test outcomes and socio-economic and socio-cultural variables were observed with the exception of the property of housing in the 2010 5ESG and in MSA, in the 2010 1HSG. As for the demographic and family variables, negative and significant correlations were observed between student’s age, the number of siblings, the place occupied by the student among his siblings (only in 2010 1HSG), the number of family members (only in 2010 5ESG), the existence of crowding and promiscuity (only in 2010 1HSG). School-age children whose parents were married obtained higher scores in LSA and MSA, but differences were significant only for MSA in both grades.

**Table 1:** Correlation coefficients between scholastic achievement (SA) in the Education Quality Measurement System (2009 SIMCE) tests of language (LSA) and mathematics (MSA) (dependent variables) and psychological, nutritional, cardiovascular risk, socio-economic, socio-cultural, demographic, family, and educational indicators (independent variables) by grade.

Independent variables	Grade							
	2010 5ESG (*n* = 682)				2010 1HSG (*n* = 671)			
	LSA		MSA		LSA		MSA	
	*r*	*p value*	*r*	*p value*	*r*	*p value*	*r*	*p value*
**Psychological variables**								
Intellectual ability (Raven’s grades)	0.463	.0001	0.559	.0001	0.469	.0001	0.587	.0001
**Prenatal nutritional background and early nutritional measurements**								
Birth weight (g)	-0.030	.5646	0.024	.6373	0.005	.9320	0.044	.4345
Birth length (cm)	-0.034	.5089	0.020	.6941	0.076	.1773	0.064	.2550
Breastfeeding duration (mo)	-0.082	.1264	-0.113	.0364	-0.038	.5109	-0.009	.8718
**Postnatal nutritional background and brain development measurement**								
Height-for-age Z score	0.108	.0100	0.155	.0002	0.093	.0317	0.204	.0001
Head circumference-for-age *Z*-score	0.159	.0002	0.242	.0001	0.173	.0001	0.330	.0001
**Current nutritional status**								
Z-BMI (Peso/Talla^2^)	-0.011	.7811	0.011	.7892	-0.124	.0019	-0.065	.1358
**Cardiovascular risk factors**								
Abdominal obesity (grades)	-0.021	.6230	-0.009	.8313	-0.185	.0001	-0.179	.0001
Systolic blood pressure (grades)	0.009	.8387	-0.076	.0973	-0.167	.0006	-0.194	.0001
Diastolic blood pressure (grades)	-0.012	.7934	-0.016	.7248	-0.142	.0035	-0.140	.0041
Tobacco (number of cigarettes per day)	-	-	-	-	-0.070	.1106	-0.120	.0061
Cardiovascular risk factors index (number)	-0.051	.2653	-0.084	.0662	-0.199	.0001	-0.182	.0002
**Socio-economic and socio-cultural**								
SES (grades)	0.295	.0001	0.305	.0001	0.291	.0001	0.316	.0001
Paternal schooling (y)	0.327	.0001	0.299	.0001	0.254	.0001	0.296	.0001
Maternal schooling (y)	0.354	.0001	0.377	.0001	0.275	.0001	0.292	.0001
House-hold head schooling (y)	0.320	.0001	0.308	.0001	0.248	.0001	0.269	.0001
Paternal occupation (grades)	0.318	.0001	0.323	.0001	0.262	.0001	0.339	.0001
Maternal occupation (grades)	0.239	.0001	0.210	.0001	0.185	.0001	0.201	.0001
House-hold head occupation (grades)	0.331	.0001	0.334	.0001	0.308	.0001	0.343	.0001
Quality of housing (grades)	0.256	.0001	0.271	.0001	0.259	.0001	0.305	.0001
Property of housing (grades)	0.008	.8516	0.007	.8686	0.091	.0336	0.063	.1456
Family stability (married)	0.049	.2387	0.093	.0259	0.073	.0881	0.098	.0229
**Demographic variables and family variables**								
Student’s age (y)	-0.140	.0007	-0.168	.0001	-0.086	.0407	-0.131	.0018
Menarche age (y)	0.014	.8475	0.029	.6803	0.041	.4907	0.076	.2035
Number of siblings	-0.102	.0140	-0.076	.0690	-0.104	.0156	-0.117	.0065
Place between siblings	-0.033	.4339	-0.042	.3239	-0.114	.0088	-0.115	.0078
Number of family members	-0.106	.0110	-0.090	.0307	-0.061	.1564	-0.034	.4244
Crowding (persons/bedroom)	-0.118	.0048	-0.110	.0089	-0.190	.0001	-0.124	.0039
Promiscuity(persons/bed)	-0.069	.1007	-0.077	.0664	-0.127	.0032	-0.141	.0010
**Educational system variables**								
EESA (grades)	0.424	.0001	0.469	.0001	0.534	.0001	0.539	.0001
School infrastructure index	0.390	.0001	0.400	.0001	0.490	.0001	0.491	.0001
L teacher’s academic background index	0.006	.9157	-		0.283	.0001	-	
M teacher’s academic background index		-	0.008	.8922	-		0.010	.8663
L teaching methodologies index	0.154	.0055	-		0.071	.2355	-	
M teaching methodologies index	-		0.207	.0002	-		0.240	.0001

Note. Pearson and Spearman correlation coefficients were used for continuous and categorical variables, respectively. EESA, level of SA of the educational establishments; L, language; M, mathematics; SES, socio-economic status; Z-BMI, body mass index Z-score.

With regard to the variables related to the educational system, positive correlations were observed between 2009 SIMCE outcomes and the SA level of the educational establishments in the 2009 SIMCE and school infrastructure in both grades and tests (*p* < .0001).

The teacher’s academic background was comparable in the different types of schools since all had a professional degree but only 16% had post graduate studies. As a consequence, only in 2010 1HSG L teachers academic background positively correlated with LSA (*p* < .0001) and only in 2010 5ESG, L teaching methodologies positively correlated with LSA (*p* = .0055). Although for M teacher’s academic background was not associated with MSA, positive correlations were found between M teaching methodologies and MSA both in 2010 5ESG (*p* = .0002) and 1HSG (*p* < .0001).

### Multiple regression analysis between LSA and MSA in the 2009 SIMCE (dependent variables) and most relevant independent variables in school-age children of the 2010 5ESG

The multiple regression models considered all independent variables significantly associated with the dependent variables, LSA and MSA. The stepwise procedure, as we explained previously in the statistical analysis, chooses those independent variables significantly associated with LSA and MSA. Multiple regression models between LSA and MSA in the 2009 SIMCE test (dependent variables) and the independent variables choose for the statistical procedure in school-age children from 2010 5ESG (Table 2) revealed that for LSA, the level of SA of the educational establishments in the 2009 SIMCE tests (high), sex (females), paternal schooling (≥ 14y), head circumference-for-age Z-score (> 0) and intellectual ability (grades I and II) were the independent variables associated with LSA results (*R*^*2*^ = .354; *p* < .0001). For MSA, the level of SA of the educational establishments in the 2009 SIMCE tests (high), head circumference-for-age Z-score (> 0), intellectual ability (grades I and II) and paternal schooling (≥ 14y) were the independent variables entered in the statistical regression model (*R* ^*2*^ = .463; *p* < .00001).

**Table 2:** Multiple regression models between language (LSA) and mathematics (MSA) scholastic achievement (SA) in the Education Quality Measurement System (2009 SIMCE) tests (dependent variables) and most relevant independent variables in school-age children of the 2010 5ESG.

Independent variables entered in the statistical regression model	Coefficients	Standard Error	*t* de Student	P>| *t* |	[95% confidence interval]	
**LSA**						
Level of SA of the educational establishments						
Ref: Medium						
High	23.044	4.950	4.660	0.000	13.320	32.769
Low	-19.217	5.384	-3.570	0.000	-29.796	-8.639
Sex						
Ref: Males						
Females	10.841	3.908	2.770	0.006	3.163	18.520
Paternal schooling						
Ref: <14y						
≥ 14y	12.597	4.560	2.760	0.006	3.638	21.555
Anthropometric measurements of nutritional background and brain development						
Head circumference-for-age *Z*-score						
Ref: < 0						
≥ 0	8.813	4.077	2.160	0.031	0.803	16.824
Intellectual ability						
Ref: Grade III						
Grade I	42.510	6.827	6.230	0.000	29.097	55.923
Grade II	22.425	4.993	4.490	0.000	12.615	32.236
Grade IV	-10.674	5.480	-1.950	0.052	-21.440	0.093
Grade V	-33.822	9.800	-3.450	0.001	-53.075	-14.568
Constant	245.022	5.332	45.950	0.000	234.546	255.499
*R*^*2*^ = .354; Root MSE = 43.468; *F* (9,496) = 30.16; *p > F* = .00001						
**MSA**						
Level of SA of the educational establishments						
Ref: Medium						
High	23.117	4.649	4.970	0.000	13.984	32.251
Low	-23.990	5.050	-4.750	0.000	-33.913	-14.067
Anthropometric measurements of nutritional background and brain development						
Head circumference-for-age *Z*-score						
Ref: <0						
≥0	15.034	3.828	3.930	0.000	7.513	22.554
Intellectual ability						
Ref: Grade III						
Grade I	52.509	6.369	8.240	0.000	39.995	65.023
Grade II	23.857	4.700	5.080	0.000	14.623	33.092
Grade IV	-23.212	5.176	-4.480	0.000	-33.381	-13.043
Grade V	-39.597	9.033	-4.380	0.000	-57.345	-21.849
Paternal schooling						
Ref: <14y						
≥ 14y	8.619	4.304	2.000	0.046	0.162	17.076
Constant	244.120	4.641	52.600	0.000	235.001	253.238
*R*^*2*^ = .463; Root MSE = 40.874; *F* (8, 497) = 53.61; *p > F* = .00001						

Note: *n* = 682; 5ESG = 5^th^ grade of elementary school; Intellectual ability grades = Grade I = superior; Grade II = above average; Grade III = average; Grade IV = below average; Grade V = intellectually defective.

### Multiple regression analysis between LSA and MSA in the 2009 SIMCE (dependent variables) and most relevant independent variables in school-age children of the 2010 1HSG

In school-age children from 2010 1HSG (Table 3), the level of SA of the educational establishments in the 2009 SIMCE tests (high), maternal schooling (≥ 14y), and intellectual ability (grades I and II) were the independent variables more relevant associated with LSA in the 2009 SIMCE (*R* ^*2*^ = .3892; *p* < .00001). In MSA, the level of SA of the educational establishments in the 2009 SIMCE tests (high), maternal schooling (≥ 14y), sex (males) and intellectual ability (grades I and II), were the independent variables entered in the statistical regression model (*R* ^*2*^ = .500; *p* < .00001).

**Table 3:** Multiple regression models between language (LSA) and mathematics (MSA) scholastic achievement (SA) in the Education Quality Measurement System (2009 SIMCE) tests (dependent variables) and most relevant independent variables in school-age children of the 2010 1HSG.

Independent variables entered in the statistical regression model	Coefficients	Standard Error	*t* de Student	P>| *t* |	[95% confidence interval]	
**LSA**						
Level of SA of the educational establishments						
Ref: Medium						
High	31.239	4.311	7.250	0.000	22.769	39.710
Low	-11.619	5.090	-2.280	0.023	-21.620	-1.617
Maternal schooling						
Ref: <14y						
≥14 y	-7.762	4.094	-1.900	0.059	-15.806	0.283
Intellectual ability						
Ref: Grade III						
Grade I	27.443	7.476	3.670	0.000	12.755	42.132
Grade II	10.390	4.358	2.380	0.018	1.827	18.953
Grade IV	-25.731	4.506	-5.710	0.000	-34.583	-16.878
Grade V	-40.746	9.518	-4.280	0.000	-59.446	-22.047
Constant	272.507	5.278	51.630	0.000	262.136	282.877
*R*^*2*^ = .389; Root MSE = 37.981; *F* (7, 499) = 45.43; *p > F* < .00001						
**MSA**						
Level of **SA** of the educational establishments						
Ref:Medium						
High	27.173	4.328	6.280	0.000	18.669	35.676
Low	-15.318	5.117	-2.990	0.003	-25.372	-5.264
Maternal schooling						
Ref: <14y						
≥ 14 y	-10.633	4.114	-2.580	0.010	-18.715	-2.551
Sex						
Ref: Males						
Females	-17.810	3.459	-5.15	0.000	-24.60577	-11.01296
Intellectual ability						
Ref: Grade III						
Grade I	33.044	7.450	4.440	0.000	18.407	47.681
Grade II	22.404	4.348	5.150	0.000	13.862	30.946
Grade IV	-32.089	4.573	-7.020	0.000	-41.074	-23.105
Grade V	-51.268	9.381	-5.470	0.000	-69.698	-32.838
Constant	298.144	5.592	53.320	0.000	287.158	309.130
*R*^*2*^ = .500; Root MSE = = 38.094; *F* (8, 500) = 61.92; *p > F* < .00001						

Note: *n* = 671; 1HSG = 1^st^ grade of high school; Intellectual ability grades = Grade I = superior; Grade II = above average; Grade III = average; Grade IV = below average; Grade V = intellectually defective.

### Association between 2009 SIMCE with 2013 SIMCE and 2017 PSU tests both LSA and MSA of school-age children of the 2010 5ESG

**Fig 5:**
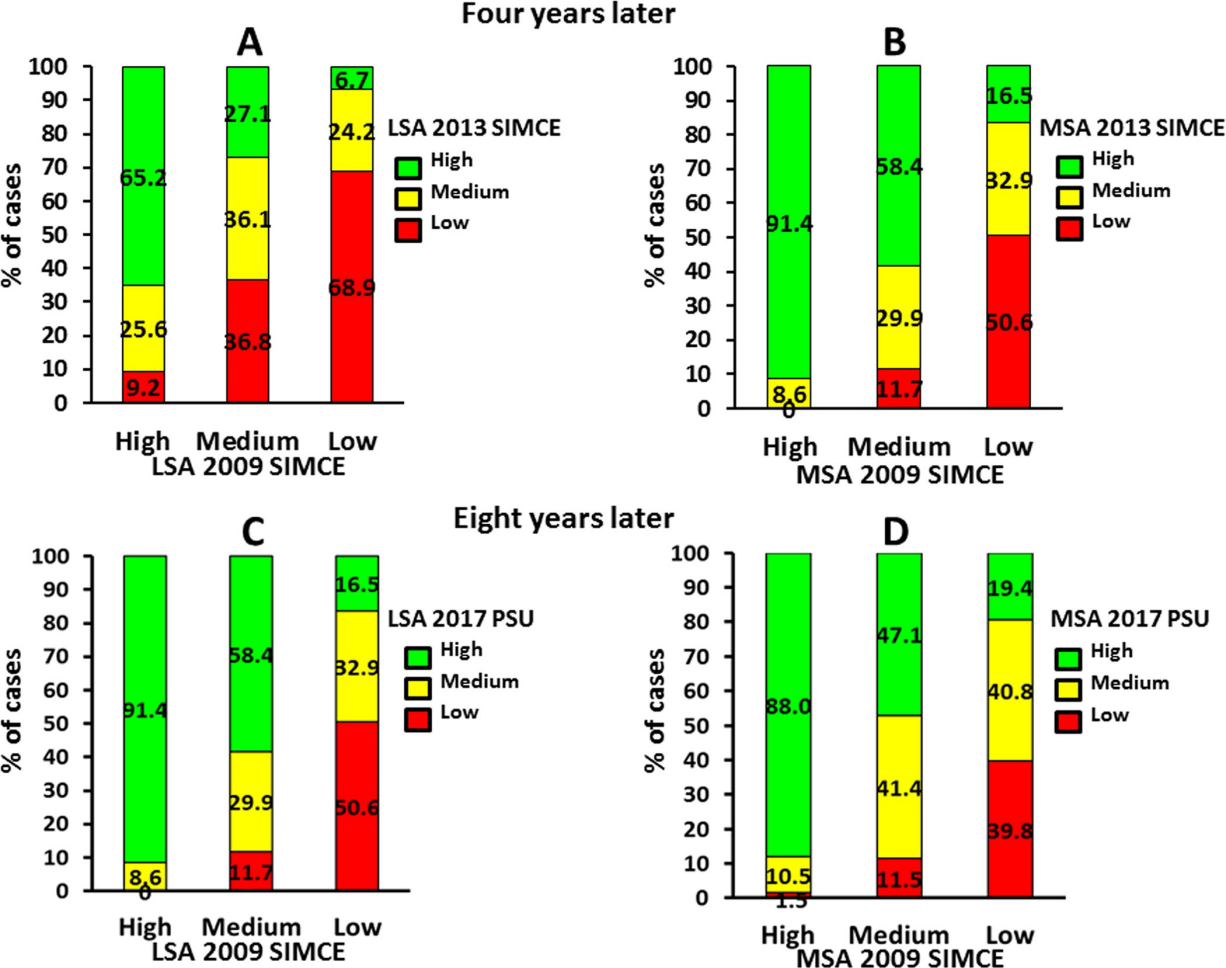
**Association between the 2009 Quality Education Measurement System (2009 SIMCE) test of school-age children of the 2010 5ESG, four years later, with the 2013 SIMCE test, when they graduated from 8**^**th**^ **grade of elementary school, both language (LSA) (A) and mathematics (MSA) (B) and, eight years later, with the 2017 University Selection Test (2017 PSU) outcomes, for university admission, when they graduated from high school, both LSA (C) and MSA (D).** Note. A. (*X*^*2*^_*o*_ (4df) = 167.580 > *X*^*2*^_*t*_ (4df) *p <* .001 = 18.465). B. (*X*^*2*^_*o*_ (4df) = 219.503 > *X*^*2*^_*t*_ (4df) *p <* .001 = 18.465). C. (*X*^*2*^_*o*_ (4df) = 116.899 > *X*^*2*^_*t*_ (4df) *p <* .001 = 18.465). D. (*X*^*2*^_*o*_ (4df) = 95.113 > *X*^*2*^_*t*_ (4df) *p <* .001 = 18.465).

Four years later, LSA and MSA in the 2009 SIMCE of school-age children of the 2010 5ESG were found to be positively associated with LSA (A) and MSA (B) in the 2013 SIMCE when they graduated from 8^th^ grade of elementary school (*p* < .001 and *p* < .001, respectively) (Fig 5). This was also observed, eight years later, with LSA (C) and MSA D), in the 2017 PSU for university admission, when they graduated from high school (*p* < .001 and *p* < .001, respectively).

### Association between 2009 SIMCE and 2013 PSU tests both LSA and MSA of school-age children of the 2010 1HSG

**Fig 6:**
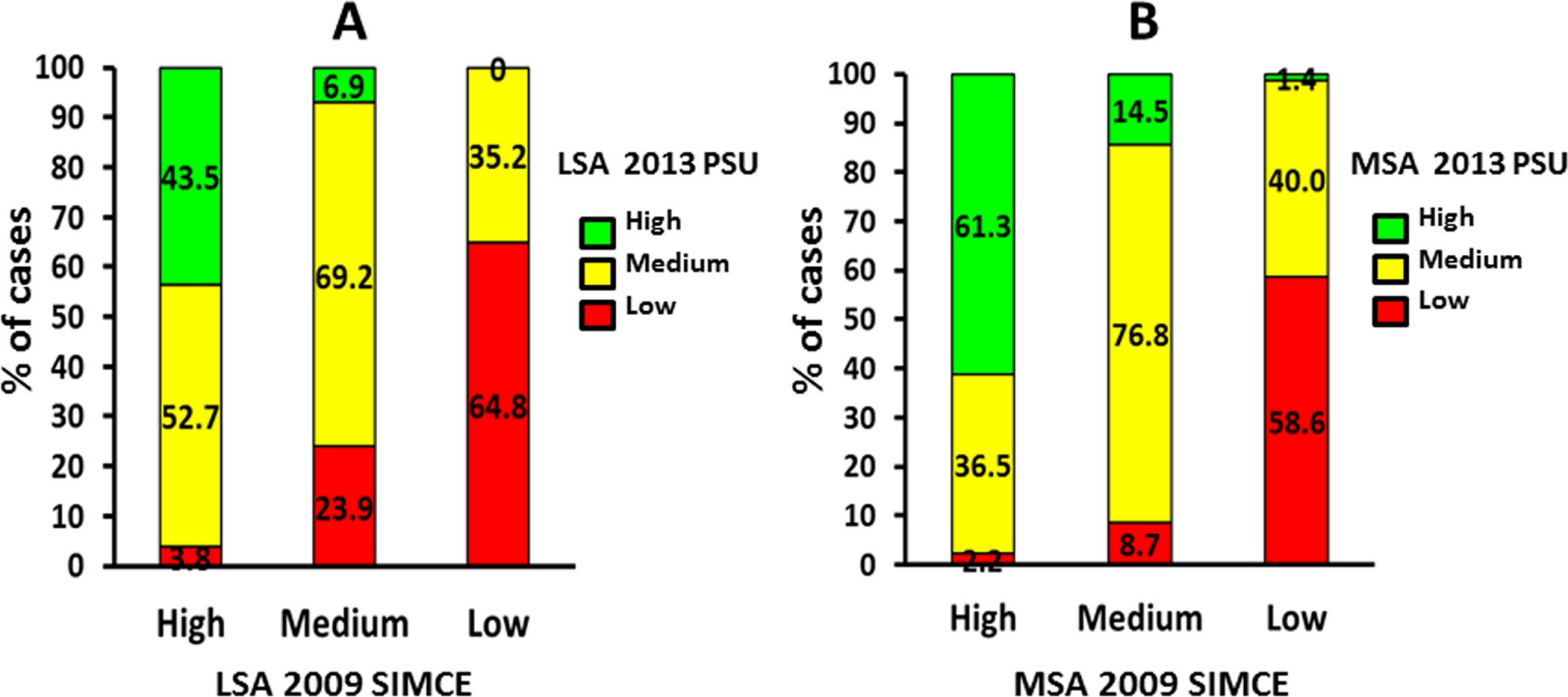
**Association between the 2009 Quality Education Measurement System (2009 SIMCE) test of school-age children of the 2010 1HSG, four years later, with the 2013 University Selection Test (2013 PSU) outcomes, for university admission, when they graduated from high school, both language (LSA) (A) and mathematics (MSA) (B).** Note. LSA: (*X*^*2*^_*o*_ (4df) = 195.763 > *X*^*2*^_*t*_ (4df) *p <* .001 = 18.465). MSA: (*X*^*2*^_*o*_ (4df) = 291.332 > *X*^*2*^_*t*_ (4df) *p <* .001 = 18.465).

Similarly, LSA and MSA in the 2009 SIMCE of school-age children of the 2010 1HSG were found to be positively associated with their 2013 PSU outcomes both LSA (A) and MSA (B), which they took at the end of high school for admission to university (*p* < .001 and *p* < .001, respectively) (Fig 6).

## Discussion

The findings of this study reveal, that the level of SA of the educational establishments in the 2009 SIMCE tests to which school-age children are subjected, the intellectual ability, parental schooling, head circumference and sex were the variables significantly associated in the statistical regression model, with their SA. These results were also observed in the sample of 2010 1HSG students when they graduated from high school four years later and took the 2013 PSU [56].

### The level of SA of the educational establishments

The level of SA of the educational establishments in the 2009 SIMCE tests, an educational system variable, was a significant predictor of 2009 SIMCE outcomes in the school-age children, and was found to be significantly associated with the type of school attended and the SES of the family. Therefore, school-age children from high and medium SES levels studied mainly in the educational establishments with high levels of SA in the 2009 SIMCE tests. Socio-economic disadvantage appears to entrench the educational disadvantage in Chile, with SES reflecting the quality of upbringing and schooling children receive, and thereby influencing their chance of attending university.

Schools with high levels of SA probably create a more stimulating environment, provide a more adequate infrastructure which favours the learning process and the parents themselves have higher levels of education and income. Students attending these schools develop higher levels of intellectual ability, have higher head circumference-for-age Z-scores, and are trained by teachers with better academic backgrounds who apply more efficient teaching methodologies [76,77].

### Intellectual ability

Intellectual ability was one of the best predictors of SA and is explained to a significant point by maternal intellectual quotient, and by brain volume and nutritional status during the first year of life [7,13,20,35]; this has been observed independently of age, sex and socio-economic stratum [35]. Results from other studies carried out in Chilean school-age children to determine the interrelationships between intellectual ability and socio-economic, cultural, family, mass media exposure, demographic and educational factors showed that maternal schooling was the variable with the greatest explanatory power in intellectual ability variance [78]. These results are in agreement with other investigators who have emphasized that children’s intelligence is significantly associated with SA, maternal schooling, maternal intelligence, head circumference and brain volume as well as antecedents of undernutrition in the first year of life [2,3,7,11–14,20,21,34–36,42,49–51].

### Parental schooling level

Paternal and maternal schooling levels were the socio-economic and socio-cultural family variables which more accurately predict 2009 SIMCE outcomes as well as the results of intelligence testing in 2010 5ESG and 1HSG, respectively. This reflects the widely acknowledged opinion that intelligence is the best predictor of SA, as shown in this study, since brighter parents attain higher levels of education, occupy better-paying jobs and form families with higher incomes [7,11–14,17,20]. It has been described that children from less advantaged families were up to twice as likely to be in the lowest quintile of mathematics and literacy scores. Around two-thirds of this elevated risk was ‘direct’ and the majority of the remainder was mediated by early cognitive ability and not self-regulation [79]. Maternal schooling is the strongest predictor of long-term SA and cognitive neurodevelopment in their children even among disadvantaged groups since, as previously stated, they are the main source of intellectual stimulation; maternal intelligence has been shown to be the best predictor of a child’s intelligence [7,11,12,14,21,22–25,36,80].

### Prenatal and postnatal nutritional background and current nutritional status

Head circumference, the anthropometric indicator of nutritional background and brain development was another important predictor of 2009 SIMCE outcomes in 2010 5ESG school-age children in both tests. This was not observed in school-age children from 2010 1HSG probably because a proportion of the deprived children (head circumference-for-age Z-score <0) had dropped out along these school years [41]. Head circumference has been defined as the most important anthropometric parameter associated with 2009 SIMCE outcomes [6] and agrees with other studies that stated that this is the most relevant anthropometric parameter, significantly associated with SA, parental schooling levels and especially with maternal schooling and intelligence: head circumference is positively and highly correlated with brain volume [2–7,11–14,22,35,41,81]. Moreover, school dropout rates relate with head circumference but not with weight or height [41].

Birth weight and birth length, reflect the mother's nutritional conditions, besides being a predictor of later child growth [10,11]. Results from several authors reported that low birth weight, birth length and breastfeeding infants are at greater risk for SA and intelligence at school age although other investigators did not find any relationship study [4,5,11,12,20,34–40]. In the current study, prenatal nutritional background and early nutritional measurements, were not found to be significantly associated with 2009 SIMCE outcomes and this does not agree with the results of other investigators [34]; the exception to these results was the negative correlation found for breastfeeding and MSA in 2010 5ESG school-age children. This could be explained because breastfeeding duration tends to be longer in families of the low SES in whom SA is significantly lower than in families of the high SES [82]. Although some authors report significant associations between breastfeeding, cognition and brain size, others emphasize that this association can be explained largely by socio-demographic factors, parental lifestyles and maternal intelligence [40,82–85].

### The impact of sex

Sex differences also contribute to explain the 2009 SIMCE outcomes in LSA only in school-age children of the 2010 5ESG (favouring females) and in MSA of school-age children from 2010 1HSG (favouring males). Our results are in agreement with those of other authors, although, as stated previously, recent data suggest that in many countries this gap is closing [31–33].

### Dietary intake habits, physical activity and cardiovascular risk factors

Some independent variables such as dietary intake habits and physical activity were previously tested as predictors for 2009 SIMCE outcomes but they did not contribute to explain the 2009 SIMCE results both LSA and MSA [86]. For Z-BMI a negative correlation was found only in LSA of school-age children from 2010 1HSG and some publications show an inverse and significant association between SA and obesity [43–46]; nevertheless, other results suggest that obesity is not associated with academic performance [4,5,12,47,48]. Our recent findings in an eight-year follow-up study also show that obesity and SA in the SIMCE and PSU tests, both with nationally coverage in Chile, are not significantly associated [87]. Results also suggest that physical fitness in boys and obesity status in girls could be important factors not only for health status but also for SA, independent of socio-economic and behavioural backgrounds [45].

Cardiovascular risk factors negatively and significantly correlated with 2009 SIMCE outcomes only in school-age children of 2010 1HSG and in both 2009 SIMCE tests, with the exception of smoking, although these results did not contribute to explain the results. Other findings do not support the existence of consistent associations between blood pressure and subsequent performance in tests assessing various cognitive domains in adolescents [88].

### Limitations of study

A limitation of the present study is that genetic and environmental factors that have been described by some authors as significant determinants for SA, intellectual ability, head circumference with brain size and other determinant variables establish complex interactions which were not measured in the present study and for these reasons further research is necessary [89–91].

### Recommendations for future research

Our results suggest that significant improvements in the quality of education are positively and significantly associated with improvements in all national socio-economic sectors of activity because education is a multifactorial problem influenced by economic, socio-cultural, family, nutritional status, psychological and educational system variables among others.

The purpose of the present investigation is to contribute to increase the existing evidence for the formulation of a theory about SA, which is difficult to establish, since its determinants vary from child to child [21]. The present study represents one of the most extensive explorations carried out in Chile focussing on the assessment of the determinants of SA. This evaluation is unique in terms of the range and number of variables measured and the combination of child (nutritional, intellectual, brain development, cardiovascular risk factors, socio-economic, demographic and educational characteristics), family and educational system factors. In summary, the level of SA of the educational establishments in the 2009 SIMCE tests, intellectual ability, paternal and maternal schooling levels, head circumference and sex, are important predictors of 2009 SIMCE outcomes. These findings confirm the hypothesis that intellectual ability, the level of SA of the educational establishments in the 2009 SIMCE tests, sex, parental schooling levels and head circumference-for-age Z-score are the most relevant parameters associated with SA. The R-squared values were higher for MSA (46 to 50%, in school-age children of the 2010 5ESG and 1HSG, respectively) than LSA (35 to 39%, respectively). This can be explained because mathematics requires higher order thinking skills that need larger brain size and intellectual skills, since in both grades correlations between MSA and both intellectual ability and head circumference-for-age Z-score were higher than those registered with LSA; however, more research is needed to explore the extent to which brain structures are associated with learning [56]. Secondly, the findings of this study also reveal that 2009 SIMCE outcomes are a good predictor of 2013 SIMCE results in school-age children of the 2010 5ESG and, of PSU outcomes in both grades.

The results of this study should be considered as showing a statistical association and do not represent a direct cause and effect relationship. In Chile, SIMCE, a test with national coverage is an excellent predictor for the results in later SIMCE tests including the PSU scores, for university admission. These results also have clinical implications, since they allow the identification of risk groups with suboptimal learning, in order to implement all necessary measures to improve their outcomes starting from an early age. These findings may be useful in planning public policies in the education and health sectors in this and other countries.

## Supporting information

S1 Dataset Total.zip. This database includes the variables considered in this study (Excel).
(ZIP)

## Abbreviations

1HSG: 1^st^ grade of high school
5ESG: 5^th^ grade of elementary school
BMI: body mass index
L: language
LSA: language scholastic achievement
M: mathematics
MSA: mathematics scholastic achievement
PSU: University Selection Test
SA: scholastic achievement
SES: socio-economic status
SIMCE: Quality Education Measurement System
